# Perceived supports and evidence-based teaching in college STEM

**DOI:** 10.1186/s40594-019-0166-3

**Published:** 2019-04-09

**Authors:** Meghan E. Bathgate, Oriana R. Aragón, Andrew J. Cavanagh, Jonathan K. Waterhouse, Jennifer Frederick, Mark J. Graham

**Affiliations:** 1 Poorvu Center for Teaching & Learning, Yale University, 301 York Street, New Haven, CT 06511 USA; 2 College of Business, Clemson University, 170 Sirrine Hall, Clemson, NC 29634 USA; 3 Educational Alliance, 344 E 14th St, New York, NY 10003 USA; 4 Department of Ecology and Evolutionary Biology, Yale University, 165 Prospect St, New Haven, CT 06511 USA

**Keywords:** Evidence-based teaching, Teaching supports, Teaching barriers, College STEM

## Abstract

**Background:**

Evidence-based teaching, such as active learning, is associated with increases in student learning and engagement. Although many faculty are beginning to adopt innovative practices, traditional lecture-based teaching tends to dominate college science education. What are the factors associated with faculty’s decision to incorporate evidence-based teaching? While there are known *barriers* that limit adoption of evidence-based practices in science classrooms (e.g., lack of time, student resistance), the present work reveals that instructors’ perceptions of *supports* (e.g., access to teaching resources, encouragement from colleagues) shows a stronger relationship to instructors’ use of evidence-based teaching.

**Results:**

These results come from a uniquely large dataset of college science faculty and instructors from across the USA (*n* = 584), who received training in evidence-based teaching. Multiple linear regression analyses of the relationship among perceived supports, barriers, and reported implementation of evidence-based practices showed that instructors report greater implementation when they perceive more social, personal, and resource supports even when barriers are also indicated as present.

**Conclusion:**

Faculty’s perceived supports, not perceived barriers, are most strongly related to their reported implementation of evidence-based teaching. These findings suggest relevant stakeholders devote increased attention identifying and building the factors that promote evidence-based teaching in addition to reducing what inhibits it.

**Electronic supplementary material:**

The online version of this article (10.1186/s40594-019-0166-3) contains supplementary material, which is available to authorized users.

## Background and literature review

Evidence-based teaching (EBT) involves educational practices derived from empirical data that show a well-established association with improved course grade, student feedback, and course-driven learning goals (Cavanagh et al., 2016; Freeman et al., 2014; Gross, Pietri, Anderson, Moyano-Camihort, & Graham, 2015; Wieman, 2014). EBT encompasses a variety of practices, including active learning and student-centered approaches encouraging students to interact with class content in a more constructivist way (Greeno & Engeström, 2014; Vygotsky, 1978), often building their knowledge through inquiry-based learning, open-ended problems, group work, discussions, and reflection.

The need for evidence-based teaching in college classrooms is increasingly clear as research shows a rigid and traditional (e.g., purely lecture based) classroom approach and can systematically marginalize students, even unintentionally, through the structure and assessments used in the classroom and the cultural norms of science (Basile & Lopez, 2015; Moss-Racusin, Dovidio, Brescoll, Graham, & Handelsman, 2012). This pattern seems to be especially true in STEM disciplines where large introductory courses are generally lecture based with little scaffolding or support for students who are often in their early years of college (Stains et al., 2018). In a 2014 meta-analysis, Freeman et al. showed increases in learning and decreases in failure rates for students in active learning classes (i.e., students who experienced a student-centered constructivist pedagogy) compared to traditional lecture-style classrooms. Freeman and others (Association of American Universities Undergraduate STEM Initiative, 2013; Wieman, 2017) have argued that innovation to college science instruction based on practices that are grounded in evidence is necessary for improving learning and equity in STEM education, particularly in light of the evidence that traditional lecture is associated with a 55% increase in failure rate compared to evidence-based teaching (Freeman et al., 2014).

Driven by this empirical work, there are broad-sweeping national calls to introduce evidence-based teaching into the sciences where introductory courses are often large, lecture-based, and teacher-centered (Bradforth et al., 2015; Handelsman et al., 2004; National Research Council, 2003; Pfund et al., 2009; President’s Council of Advisors on Science and Technology, 2012). Nevertheless, large-scale transformation of science education lags behind this call to action. Recent large-scale observational work shows that STEM faculty continue to frequently use traditional lecture practices (Stains et al., 2018). Even when faculty are well-trained in EBT, there is strong variation in how fully they incorporate EBT into their practice (Aragón, Dovidio, & Graham, 2017; Fairweather & Paulson, 2008; Lazerson, Wagner, & Shumanis, 2000).

What accounts for this variation in implementation? Individuals’ teaching choices arise from a mixture of personal and contextual factors. Ajzen’s theory of planned behavior (Ajzen, 1985, 2011) captures these elements well, outlining the importance of personal attitudes, subjective norms of a given context, and perceived control or self-efficacy, towards a behavior as drivers of behavioral change. In relation to implementing EBT in STEM classrooms, the theory of planned behavior encompasses important features of faculty’s teaching choices. This includes faculty’s personal attitudes towards EBT, the social norms within faculty’s specific academic departments around others’ perception and emphasis around this teaching approach, and faculty’s self-efficacy in finding or developing EBT resources and using them effectively. Factors such as these are often included in work related to faculty’s implementation of EBT (Brew & Mantai, 2017; Hora, 2012; Lee, 2007; Lund & Stains, 2015). Our work reflects this framework as we examine how faculty perceive the personal, social, and academic factors within their teaching environment as supportive or prohibitive of behavioral change towards incorporating EBT.

Within a given institutional context, there are factors that promote or decrease the degree to which faculty incorporate EBT into their courses. To date, the literature has overwhelmingly focused on the existence of *barriers*, such as the perception of departmental and logistic constraints, as a source for variation in faculty’s use of EBT. For example, faculty frequently discuss time constraints as a major limiting factor, both within and outside the course. Within the course, faculty refer to constraints of “covering” a breadth of required content (e.g., Henderson & Dancy, 2007), thus limiting more inquiry-based and group activities. Outside of the course, faculty note that their efforts for research are often more rewarded than that of teaching, therefore limiting their preparation time for developing, assessing, and revising new activities (e.g., Brownell & Tanner, 2012; Hora, 2012; Michael, 2007).

Faculty’s perception of student reluctance to participate in active learning due to the lack of student preparation and variation in students’ past experience with EBT also serves as a barrier, as does faculty’s concern that violating students’ expectation for traditional lecture may negatively impact their student evaluations (Herreid & Schiller, 2013; Michael, 2007). It is clear these barriers extend well beyond the classroom context and into institutional considerations of promotion, research, and expectations as a scientist (Bradforth et al., 2015; Brownell & Tanner, 2012), many of which are beyond an individual faculty member’s control.

Conversely, there are factors that faculty say encourage, or *support*, the incorporation of EBT into their classroom. There is growing emphasis towards these perceived supports and their relationship to improving teaching in higher education STEM courses (Association of American Universities Undergraduate STEM Initiative, 2013; Wieman, 2017). Much of this work has used qualitative analyses including focus groups, case studies, and in-depth projects to define the supports that promote faculty and departmental adoption of innovative teaching approaches. These efforts identify many overlapping themes, pointing to specific events and opportunities that support, or are hypothesized to support, the incorporation of EBT into teaching. For example, Shadle, Marker, and Earl’s (2017) recent work with 169 STEM faculty identified factors such as a department’s emphasis on teaching in tenure decisions, collaboration with communities of practice, and faculty’s desire for improved student outcomes as supportive to educational change. Other supports include professional development trainings and access to active learning classrooms (i.e., those that prohibit traditional lecture seating) (Ebert-May et al., 2011; Lattuca, Bergom, & Knight, 2014; Pfund et al., 2009; Shadle et al., 2017), or having access to knowledgeable pedagogical specialists and existing research on effective teaching (Corbo, Reinholz, Dancy, Deetz, & Finkelstein, 2016; Wieman, 2017).

Beyond specific practices, these studies point towards departmental culture, i.e., a shared set of beliefs, values, and practices towards teaching, as a major influence of EBT (Bradforth et al., 2015; Corbo et al., 2016; Henderson & Dancy, 2007; Hora, 2012; Wieman, 2017). Indeed, faculty have a sense of their departmental value towards teaching expressed through the availability, reward, and explicit emphasis on teaching approaches and outcomes. These perceptions reflect the social norms of a department, which are a major driver of behavior according to the theory of planned behavior. Through the lens of this theory, a departmental culture that values and is committed to teaching efforts would likely integrate supportive practices that emphasize teaching. For example, rewarding teaching efforts in its promotion structure, providing faculty time to devote to teaching, and providing opportunity for colleagues to discuss teaching approaches in order to encourage an environment where faculty are more familiar with and implement greater EBT (Ebert-May et al., 2011; Lattuca et al., 2014). Specifically, faculty report feeling it is easier to implement EBT when there are multiple faculty also using these techniques and are able to discuss teaching ideas with peers (Henderson & Dancy, 2007).

**Fig. 1:**
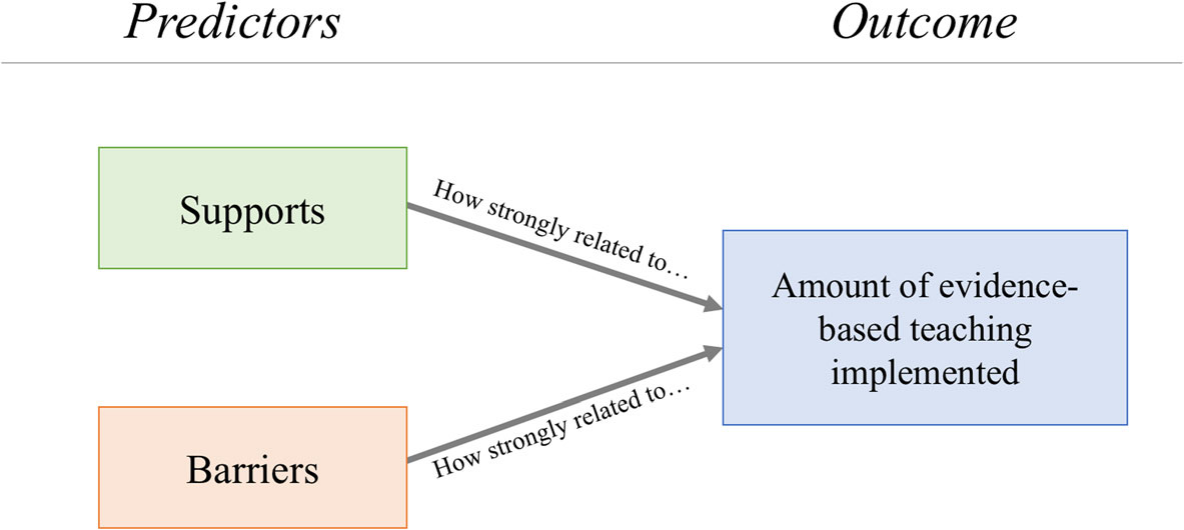
The tested model of the relationship among faculty’s perception of supports and barriers to their adoption of EBT and the amount of EBT they report using in their courses

Our efforts extend this work to examine how both perceived supports and barriers simultaneously relate to accompanying implementation data using a substantive sample of faculty from across the nation. Most of the previous work on supports and barriers unpacks faculty’s perception of these factors, but little connects these perceptions to implementation. Guided by the theory of planned behavior, we asked faculty about the personal and social factors in their teaching environment that supported or hindered their use of EBT. We then examined the empirical relationship between these factors and their reported use of EBT. Specifically, we tested the number of perceived supports and barriers and the degree to which faculty reported implementing EBT in their courses. Our major research question is *what is the relationship between faculty’s perception of supports and barriers to EBT and their reported use of EBT practices in the classroom?* See Fig. 1 for our tested model. In answering this question, we describe patterns and correlations among our variables. See the “Analysis” section for more details. We aim for our findings to have practical implications for how faculty envision the process of adopting EBT into their courses and how professional development trainings for faculty are framed.

### Study context

The data presented here come from a large sample of college science instructors from across the USA trained in EBT. All participants are alumni of the Summer Institutes for Scientific Teaching (SI), a regional week-long faculty development program that trains individuals to incorporate EBT practices (e.g., active learning, assessment, inclusive teaching, backward design) into their classrooms (Handelsman, Miller, & Pfund, 2007). Leveraging longitudinal data to enhance program impact: The Summer Institutes on Scientific Teaching; Pfund et al., 2009). Individuals applied to the SI through an application form outlining their intentions for attending the training and how they hope to apply the curriculum to their teaching. Individuals were accepted based on the capacity and staffing of the regional trainings held throughout the USA—which regularly include east coast, west coast, and mid-west locations. Each training generally included 30–45 attendees. This hands-on, group-facilitated training has been running since 2004 and has trained over 1800 instructors from over 350 institutions in the evidence behind effective teaching practices. Although this sample consists of faculty who self-selected to attend a professional teaching training, it nevertheless provides a unique look into how a large group of college science faculty perceives supports and barriers to implementing EBT.

Conceptually, Ajzen’s theory of planned behavior (Ajzen, 1985, 2011) serves as a theoretical framework for this study. Ajzen’s framework emphasizes that intention to pursue and persist in a given task is influenced by the context in which that behavior is performed. The faculty attending the SI are motivated enough to participate a training on EBT, but what ends up being incorporated in their classrooms is likely influenced by the context and social norms of their institution. As such, we focus on the contextual factors that may support or inhibit the implementation of EBT. Professional development research (e.g., Lund & Stains, 2015) and our ongoing evaluation work of the Summer Institutes provided guidance for the creation and analyses of the specific types of supports, barriers, and implementation measured here.

## Methods

### Participants and procedure

Instructors who attended the Summer Institutes between 2004 and 2014 were contacted via email to participate in the study through an online survey (*N* = 1179). The survey took approximately 15–25 min and could be completed over multiple sittings if desired, as each participant was emailed a personalized link. The initial page of the survey collected consent to participate and no compensation was given for completion. Seven hundred and twenty-eight participants responded to our survey (~ 62% response rate). For the purpose of our analyses, only faculty and instructors who taught science courses at the college level and have participated in the Summer Institutes within the past 5 years were included (*N* = 584). The decision to use participants from the past 5 years was made for two reasons. First, we wanted to capture recent changes to the SI curriculum and, second, we wanted a sample of faculty who were relatively early in their progression towards EBT. These criteria represent most SI alumni, since the target audience of the SI is faculty from college STEM departments at research intensive institutions who also teach large introductory courses. Eighty-two percent of our sample teach introductory course and 73% do so at least once a year. Teaching experience ranged from 1 to 43 years (*M* = 12.6 years; SD = 9.4 years), with participants holding appointments across a range of academic positions, including tenure track positions (62%), non-tenure track positions (28%), and a small number of postdoctoral scholars and researchers (10%). Sixty-one percent of participants are female and most are Caucasian (83%). This demographic representation is typical for SI attendees. Additionally, not everyone completed the full survey, and participants were included when they responded to the measures used in each analysis in order to maintain the greatest statistical power. This resulted in different sample sizes for some analyses (e.g., 558 of the 584 responded to implementation items). These differences are noted where appropriate and were never fewer than *N* = 465.

### Measures

#### Implementation measure

A 19-item self-report survey assessed critical aspects of the EBT practices (e.g., active learning, assessment approaches, inclusive teaching practices) taught at the SI. These items were used as a measure of faculty self-reported implementation of EBT. This measure was initially developed by reviewing the practices taught in SI curriculum (Handelsman et al., 2007), reviewing recent literature outlining observable practices related to EBT in specific relationship to the practices in the curriculum (Couch, Brown, Schelpat, Graham, & Knight, 2015; See Additional file 1 for detailed connections), and aligning items to represent the theoretical model of the SI developed through the National Science Foundation Grant Transforming Undergraduate Education in STEM (No. 1323258). These items were also reviewed by the authors and facilitators from the SI. These items included elements such as reflective practices (e.g., “Encouraging students to think about their own learning processes, aka metacognition”), group discussion (e.g., “Using exercises that generate group discussion”), and clarity of learning objectives and expectations (e.g., “Implementing formative assessments while learning is occurring that inform students’ progress towards a desired outcome”).

The implementation measure consisted of binary-response items asking instructors whether they implemented each practice in their courses. This measure was part of a matrix checklist that asked faculty about their perspectives and use of each practice taught at the SI. Each row of the matrix was one of the 19 implementation practices. The checklist columns asked faculty whether they were exposed to each practice at the SI followed by a series of questions about their “buy-in” to each practice (e.g., whether they thought it was a good idea, whether it was compatible with their teaching style). The last column of the matrix was the checklist stating “I have implemented this practice in my course(s).” A sum of this final column was used as our implementation measure.

The beginning instructions to this measure where as follows, “The next few questions will ask you about scientific teaching practices presented to you at the SI. In another section we will give you the opportunity to tell us about your teaching practices before you attended the SI. Please read each statement in the left column, and then please check ALL boxes that apply. These data are interpreted specifically by the boxes that you check. If you do not check a box we will assume that you do not endorse the statements related to it.” Faculty were also given an example of how to complete the measure for clarity. Three practices were presented per survey page to minimize opportunities for faculty to unintentionally omit responses to practices.

#### Perceived supports and barriers

We used an iterative process to compile, categorize, and review free-response data from 249 previous SI alumni evaluation surveys^1^, spoke with SI leaders who ran the training programs and reviewed previous literature with the purpose of developing a list of commonly stated supports and barriers. These categories can be found in Table 1. The number of items within each category reflects instructors’ responses to the free-response questions and are therefore not always equal across categories (e.g., logistic considerations has four items where academic receptivity has seven items).

**Table 1:** Categories for barriers and supports

Definition/conceptualization	Example items
Academic receptivity	
Instructor perception of support from their academic department and colleagues towards implementing evidence-based teaching	*Support:* The culture in my department appreciates effort expended for teaching. *Barrier:* My efforts in teaching could be misconstrued as reduced efforts in research and hurt my career.
Logistic	
Instructor perception of the practical ease or challenge of implementing evidence-based teaching within their class constraints	*Support:* I am able to cover all of the core material. *Barrier:* I do not have enough class space for group activities.
Student receptivity	
Instructor perception of their students’ reaction to evidence-based teaching	*Support:* My students appreciate the interactive aspect of learning. *Barrier:* My students will not appreciate having to work more in class.
Personal teaching preference	
Instructor perception of alignment between their preferred teaching techniques and evidenced-based teaching	*Support:* Having an interactive classroom is more fun for me. *Barrier:* I am not comfortable teaching in an interactive way.

An index was created from these responses and includes 30 support items and 30 barriers, each with a binary response (a “1” was given if an instructor checked that they perceived an item as a support/barrier and a “0” was given when the item was not checked). Tables 2 and 3 display each support and barrier item by category. To complete this measure, instructors were given the following directions for supports and barriers, respectively: supports, “Please indicate all benefits that you foresee or found to implementing the teaching practices presented at the Summer Institute. Please check ALL boxes that apply.” and barriers, “Please indicate all the instances below that you foresee or found to be obstacles in implementing the teaching practices presented at the Summer Institute.” A sum of support items and barrier items were independently used for our two main independent variables.

**Table 2:** Categories of supports with the percentage of instructors who perceive each item (*n* = 584)

Number	Academic	Percentage	Number	Student receptivity	Percentage
S01	My colleagues (peers) are supportive*	58	S20	My students are teaching each other*	63
S02	My department appreciates my efforts to improve scientific teaching*	51	S21	My students will cooperate with the activities*	49
S03	I get support from my department*	46	S22	My students are focused and engaged in the material*	47
S04	My colleagues (senior) are supportive*	42	S23	My students appreciate the interactive aspect of active learning*	41
S05	I get support from the SI community*	36	S24	My students who are farther along in the material are encouraged when they are in group work*	22
S06	The culture in my department appreciates effort expended for teaching*	27	S25	My students who are shy are comfortable during group work*	19
S07	The culture in my department prioritizes teaching over research and my efforts are appreciated	12	S26	My students who have a hard time focusing are on task during group work*	14
Number	Personal teaching	Percentage	Number	Logistic	Percentage
S08	I enjoy being interactive with students*	74	S27	I have been able to find materials to help me with activities*	53
S09	Having an active classroom is more fun for me*	64	S28	Clicker activities are a fun way to make my point*	45
S10	The class is transformed into a lively space during group activities*	62	S29	I am able to cover the material without a lecture*	30
S11	I enjoy coming up with class activities*	57	S30	I am able to cover all of the required core material*	24
S12	I am getting to know my students better*	57			
S13	I am comfortable giving students ongoing feedback and enjoy the interactions with them*	56			
S14	I am excited to be figuring out new activities for class*	52			
S15	I am more comfortable teaching in an interactive way*	50			
S16	I feel as though I have a handle on the process of scientific teaching*	48			
S17	Scientific teaching is my style of teaching*	44			
S18	I feel that implementing inclusive teaching is making me a more sensitive teacher*	41			
S19	I am more comfortable with an active classroom than with my lecture and PowerPoints*	32			

*Indicates the item is significantly related to implementation through a two-tailed Spearman binary correlation (*p* < .05)

**Table 3:** Categories of barriers with the percentage of instructors who perceive each item (*n* = 584)

Number	Academic	Percentage	Number	Student receptivity	Percentage
B01	The culture in my department prioritizes research over teaching	31	B16	I am concerned for my students who are shy feeling uncomfortable during group work	37
B02	My efforts in teaching could be misconstrued as reduced efforts in research and hurt my career	16	B17	My students are not as enthusiastic about active learning as I thought they would be	26
B03	My colleagues (senior) to me are not supportive	12	B18	My students will not appreciate having to work more in class	23
B04	My colleagues (peers) are not supportive*^a^	10	B19	I am worried about my students who have a hard time focusing taking the group work off task	21
B05	My department is not supportive	4	B20	I am concerned about my students who are farther behind getting discouraged when they are in group work	21
B06	The SI community has not continued to support me	2	B21	My students will not appreciate the interactive aspect of active learning	19
			B22	I am worried that my students will not cooperate with activities	15
			B23	My students are not focused enough to engage in material without more class structure	14
			B24	My students are not able to work at the level that active learning requires	9
Number	Personal teaching	Percentage	Number	Logistic	Percentage
B07	I have a hard time coming up with class activities*	26	B25	I do not have enough time to prepare class materials	58
B08	I do not feel that I have enough knowledge to implement inclusive teaching (i.e., sometimes I do not even know what the correct thing is to say)*	16	B26	I do not have enough time during class for the activities	45
B09	The whole process of redesigning my courses is simply intimidating*	13	B27	I worry that we will not be able to cover all of the required core material*	40
B10	I am overwhelmed with trying to figure out what to do and I do not know where to start*	8	B28	I do not have enough class space for group activities	30
B11	I am not enough of an extrovert to be so interactive with students*	7	B29	There is not money for class activities	15
B12	I am more comfortable with my PowerPoints as they are*	5	B30	There is no money for clickers	6
B13	I am not comfortable teaching in an interactive way*	4			
B14	I am not comfortable giving students ongoing feedback because it might spur on uncomfortable interactions	3			
B15	[Evidence-based teaching] is simply not my style of teaching	1			

*Indicates the item is significantly related to implementation through a two-tailed Spearman binary correlation (*p* < .05)

^a^Unlike other barrier items, this item is positively associated with implementation. One interpretation of this pattern would be that an instructor would not perceive their colleagues as unsupportive of EBT until he or she began implementing and discussing it with colleagues (i.e., implementation would proceed perceiving this barrier), resulting in a positive relationship between item B04 and implementation

##### Additional variables

Additional variables were included to characterize the sample, including the length of teaching experience (number of years), professional position (e.g., assistant professor, senior professor), gender (selection among female, male, do not wish to respond), and ethnicity (binary selection among a range of ethnicities). For ethnicity, responses were recoded into (1) minority status (non-Caucasian) and (0) non-minority status (Caucasian).

### Analysis

We began with an examination of the average implementation score of EBT in our sample (*M*, SD), followed by examining the number of perceived supports and barriers and testing the difference in frequency between the two via a within-sample two-tailed *t* test. Next, we explored the most and least frequently selected supports and barriers. We also ran two-tailed Spearman binary correlations to test each support and barrier item’s individual relationship to faculty’s implementation sum. Following that, we calculated the correlations within support and barrier items and visualized these correlations using D3 for Data-Driven Documents, a JavaScript library for visually displaying interactive data, to show specific item-to-item correlations and density (i.e., the number of items with which each support or barrier was at least low-to-moderately correlated). Finally, the relationship between the sum of supports and barriers to reported implementation was tested through a multiple-linear regression where the sum of supports and the sum of barriers were the independent variables and implementation score was the dependent variable. These analyses do not determine causality but describe relationships among our variables of interest. All statistical analyses were run using Statistical Software for the Social Sciences Version 22 except where noted.

## Results

### Implementation of EBT

On average, these instructors report implementing around 11 of the 19 practices indicated from the questionnaire covering topics from their Summer Institute training (*N* = 558; *M* = 11.16 of 19 practices, or 60%; SD = 4.50).^2^ Instructors show considerable variation, with some reporting implementing many practices and others fewer (as evidenced by the standard deviation of 4.5 practices). Table 4 shows the implementation items and the percentage of faculty who reported having implemented each practice. In interpreting these percentages, it is important to remember these items are self-reported and do not necessarily reflect quality or frequency but reflect faculty’s reflections on their own use of EBT.

**Table 4:** Percentage of faculty reporting having implemented each EBT practice in our implementation measure

Implementation items	Percentage
Structuring class time to include activities that engage students in their own learning	75.2
Providing feedback to students throughout the semester	72.1
Using exercises that generate group discussion	70.7
At the onset of a course telling students what they should know and be able to do upon course completion	65.6
Considering learning goals in the design of activities for the class (backward design)	64.9
Using summative assessments of learning outcomes (i.e., to measure the students’ achievement of learning goals)	64.2
Implementing formative assessments while learning is occurring that inform students’ progress towards desired outcomes	64.0
Representing science as a process of the scientific method	62.5
Setting and communicating learning goals for students for each class	56.0
Identifying students’ misconceptions so that they may be corrected	56.0
Using Blooms taxonomy which defines depths of understanding when preparing exams	55.7
Using exercises that lead students to draw their own conclusions	54.6
Encouraging students to think of science within the context of society	53.6
Choosing diverse teaching methods to optimize learning for diverse students	53.6
Encouraging students to generate class wide discussions	44.3
Implementing inclusive teaching in the classroom	41.8
Taking precautions to reduce the influence of any implicit bias that I may hold for example grading papers without knowing the identity of the student	41.1
Encouraging students to think about their own learning processes aka metacognition	38.7
Designing class content that represents the perspectives and contributions of people with different origins genders and affiliations	32.2

Implementation, as well as the supports and barrier variables below, were within a normal range of distribution according to skewness (− 0.29, − 0.06, 0.95, respectively) and kurtosis analyses (− 0.71, − 0.86, 1.0) (George & Mallery, 2010).

### Frequency of supports and barriers

With these findings in mind, we next explored instructors’ perceptions of what encourages and challenges implementation of scientific teaching, including the frequency and co-existence of the supports and barriers instructors perceive. Instructors perceive over twice the number of supports compared to barriers (*t* (489) = 23.12, *p* < .001; large effect size: *d* = 1.33) and, on average, perceive 50% of the supports (15/30 items) and 21% of the barriers listed (~ 6/30 items).

The most frequently endorsed support items include personal teaching preference items (e.g., “I enjoy being interactive with students,” 74%), and the least endorsed are specific student support items and academic receptivity (e.g., “My students who have a hard time focusing are on-task during group work,” 14%; “The culture in my department prioritizes teaching over research and my efforts are appreciated,” 12%). In the case of barriers, logistical concerns were the most common perceived challenges (e.g., “I do not have enough time to prepare class materials,” 58%) with personal teaching preferences identified as the least frequently perceived barriers (e.g., “I am not comfortable giving students ongoing feedback because it might spur on uncomfortable interactions,” 3%).

Not all supports and barriers were significantly related to faculty’s implementation score. Some items were significantly correlated with implementation and others were not. The items that were significantly related are indicated with an asterisk in Tables 2 and 3. Most supports were significantly related to faculty’s implementation score, while only a few of the barriers show a significant relationship.

### Connectivity among supports and barriers

We calculated the correlations among the perceived support and barrier items, respectively. Correlations show us the degree to which one of the perceived supports coexist with the remaining 29 other supports on the measure. The same analysis was done with the barrier items. In general, perceived supports were more correlated than the barriers (average correlation for supports *r* = 0.301 and for barriers *r* = 0.098). See Additional file 1 for specific item-to-item correlations.

**Fig. 2:**
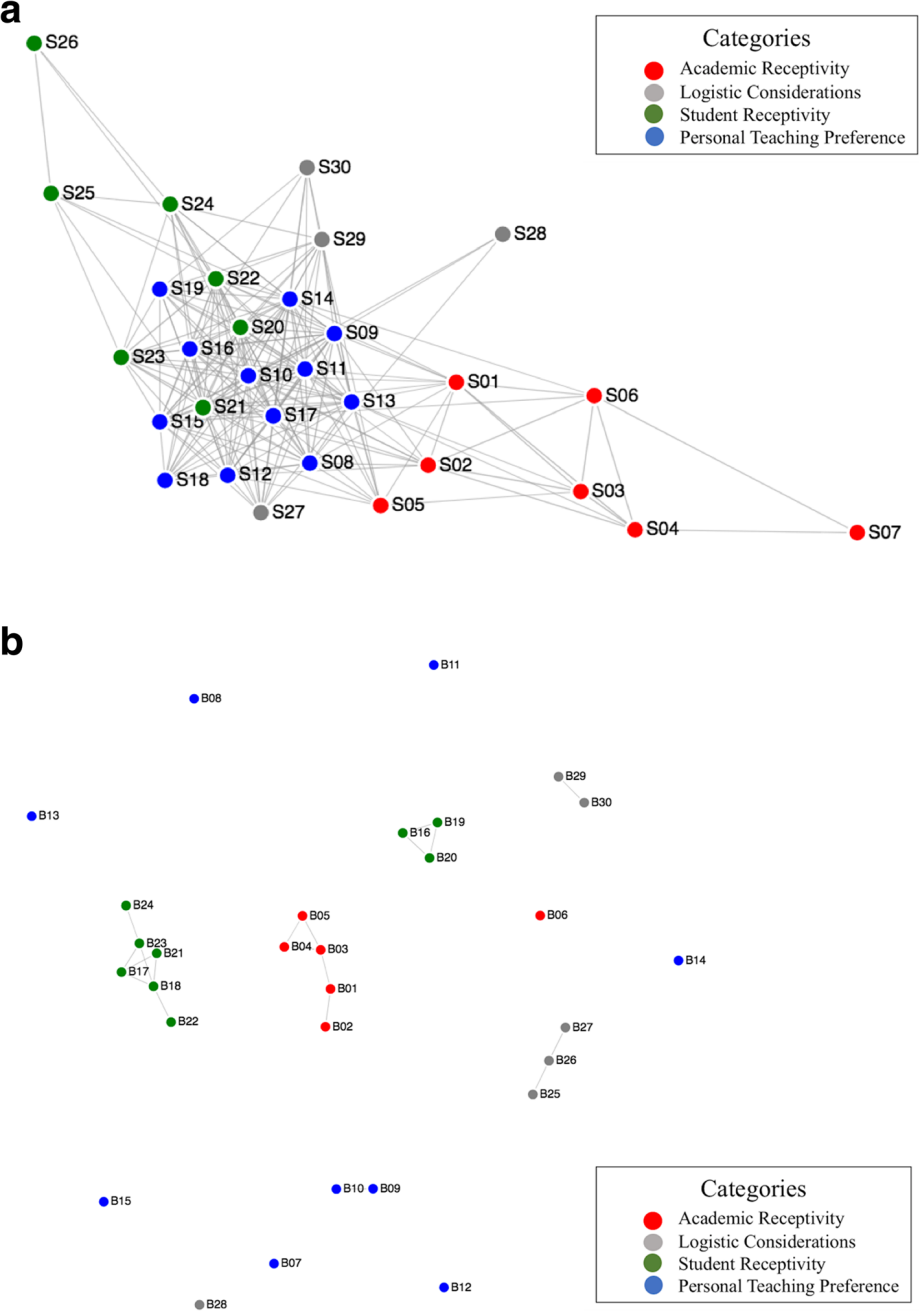
Visualization of the strongly interconnected structure of perceived supports (**a**) and the isolated structure of barriers (**b**) from 558 instructors. Each node represents a support item (**a**) or barrier item (**b**) and the color of the node represents its category (see legend)

The correlations among support and barrier items are organized in Fig. 2a and b to depict the specific connections and density of connections among items. A line is present between nodes when they have at least a low-to-moderate correlation (i.e., correlation above 0.3). Items that have more connections are centrally located to the figure and those with less connections are towards the edges. Item labels correspond to items in Tables 2 and 3. This image was made using D3 for Data-Driven Documents.

Supports (Fig. 2a) are clearly well-connected, creating a networked structure. It shows that if instructors perceived one support, they also perceived many others. No support is completely independent of any other, and 27/30 are connected with at least four others. For example, when instructors said they have access to EBT resources, they were also more likely to say that they enjoyed creating EBT activities for the students and that their students responded positively to EBT activities. Conversely, the connectivity among barriers (Fig. 2b) shows a different structure. The barriers are highly independent of each other, with 9/30 barriers not connecting to any other barrier and 11/30 connecting to only one other barrier. Instructors who perceived one barrier (e.g., having a hard time coming up activities) were not likely to perceive many others. Thus, barriers existed in much greater isolation than supports. Few barriers are well-correlated and, of those that are, few show more than a single connection with another barrier (e.g., the academic receptivity nodes).

There is also some variation in the density of connections across categories (e.g., academic receptivity, logistic considerations). Specifically, academic supports have roughly a third of the connections of personal teaching preferences, although this difference is relative as there are still over 30 links within and across this category. Teaching preferences are most strongly connected, particularly to each other. The academic support items having fewer connections show that instructors’ perception of the support they receive from their field, peers, and department is not as frequently related to other supports. This lack of relationship between academic support items and personal teaching preferences is sensible given that academic supports are less likely to be within the instructors’ direct control. Notably, there is not a uniformly clear relationship between the frequency of a support being selected and its centrality to the figure. For example, some highly endorsed supports, such as getting support from one’s department (S03), are farther from the center (i.e., less connected) than other items endorsed at a similar frequency (e.g., S17). Within the barriers, interrelationships are sparse. Of those with connections, the perception of students’ reaction to EBT are most well-connected, but only with items in that same category, and personal teaching preferences are least connected. No category connects to any other category (e.g., no student item connects to any logistic item). It should also be noted that this analysis does not determine any causal relationship but shows the frequency with which faculty perceive particular supports (or barriers) similarly.

### Relationship of supports and barriers to implementation

We first addressed whether the amount of supports and barriers instructors perceived were related to how much EBT they reported implementing. A multiple linear regression allowed us to look at how perceptions of supports and barriers simultaneously related to implementation scores. The sum of supports and sum of barriers were entered as independent variables. The amount of implementation was the dependent variable.

Table 5 displays the standardized regression betas for the model, showing that the amount of support these instructors perceived was strongly related to the reported implementation of these teaching practices. This finding is represented by the standardized beta (*β*), which shows that for every additional standard deviation in support, implementation scores increased more than half a standard deviation (*β* = 0.52) on the 19-item implementation measure. Notably, there was no relationship between perceived barriers and implementation. This pattern shows that the relationship between *supports* and implementation, as measured here, is far greater than that of *barriers* and implementation. Together, the model accounts for 27% of the variance in implementation scores (based on the *R*^2^ from the regression model)—a substantially large amount of overall reported implementation.

**Table 5:** Multiple linear regression results with reported implementation as outcome

Variable	Standardized beta
Number of perceived supports	.52***
Number of perceived barriers	− .01^NS^

Gender, ethnicity, and teaching experience (number of years) were not significant predictors of reported EBT implementation when included in regression models

*N* = 483; *R*^2^ = 0.27; *F* (2, 481) = 91.973, *p* < .0001

***p < .001; ^*NS*^not significant.

### Exploring only significant support and barrier items

Previous results exploring the relationship between individual support and barrier items showed that not all items were statistically significantly related to implementation scores (see Tables 2 and 3). As such, we did a subsequent regression with the same variables but only including the support and barrier items that were individually related to reported implementation. Since our support and barrier measures are a checklist of items not necessarily intended to be continuous, i.e., because a faculty member endorsed one support or barrier, we did not necessarily anticipate that they would select others and we wanted to see if the supports and barriers with the biggest empirical connection to the implementation outcome would demonstrate a different pattern than using all the items in the measures. For example, perhaps only a subset of barriers impacts implementation even when others are present, i.e., some barriers are present but easy to overcome.

Table 6 displays the standardized regression betas for the model, showing that supports remain most associated with reported implementation and similar to findings in Table 5. For every additional standard deviation in support, implementation scores increased almost half a standard deviation (*β* = 0.48) on the 19-item implementation measure. The sum of significant barriers was significantly associated with reported implementation but had only about a third of the strength (*β* = − 0.18). Even when the barriers most associated with reported implementation are included, perceived supports remained most related to how much EBT faculty reported implementing in their courses.

**Table 6:** Multiple regression results

Variable	Standardized beta
Subset of the number of perceived supports	.48^***^
Subset of the number of perceived barriers	− .18^***^

*N* = 557; *R*^2^ = .27; *F* (2, 555) = 104.278, *p* < .0001

***p < .001; ^*NS*^not significant

In summary, perceived supports, and not perceived barriers, related most strongly to implementation scores. The more support instructors perceived, the more evidence-based practices they reported implementing, irrespective of the barriers they perceived. Perceived supports strongly relate to implementation even when barriers are present in this sample of trained faculty.

## Discussion

Taken together, this work shows that in our sample, (1) reported implementation of evidence-based teaching (EBT) practices is greater when instructors perceive more supports, (2) the number of perceived challenges have a relatively small relationship with implementation scores, and (3) the number of perceived supports have a different (highly related) connectivity structure than perceived barriers (isolated). These findings reflect the theory of planned behavior in that the supports and barriers, which themselves reflect factors such as personal preferences and subjective norms, predict reported behavior (i.e., teaching practice) in the classroom.

The relationship between perceived supports and implementation and the lack of relationship of perceived barriers and implementation is unexpected given the typical focus on barriers as a common factor impeding implementation (Henderson & Dancy, 2007; Lund & Stains, 2015; Michael, 2007; Walczyk, Ramsey, & Zha, 2007). These findings challenge the idea that perceived barriers to EBT are the largest factor associated with EBT, at least in faculty trained in such practices.

Our data show that when faculty see resources available to them, they report implementing more EBT *despite* the continued presence of challenges. Not all barriers needed to be removed for these faculty members to implement a relatively high percentage of the 19 practices we measured. This finding echoes the work that solutions to commonly perceived barriers, such as classroom layout and course size, are not systematically tied to increases in student-centered teaching (Stains et al., 2018). Furthermore, connections among supports are strong and plentiful in this population, so that these faculty tend to see multiple supports when they perceive even one. Interventions aimed at helping faculty identify supports have the potential for a cascading effect where faculty begin to see multiple supports by uncovering or establishing a few. For example, if a faculty member identifies a colleague who is supportive of EBT, they may share resources with each other, which in turn may help the faculty member feel better prepared and enjoy their interactive classroom. Our pattern of results suggests that it would be useful for faculty to (a) identify and develop connections to academic resources, (b) identify peers who use EBT in their classrooms, and (c) develop strategies that allow them to implement even amidst existing challenges. These findings complement and provide empirical support to the recommendations of others working towards promoting EBT in college science courses (Bradforth et al., 2015; Henderson & Dancy, 2007).

This pattern also suggests that generating supports may be more efficient than the notion of dismantling barriers, as the barriers data points appeared less related to each other (i.e., reducing one does not have much effect on others). For example, as can be seen in Fig. 2b, overcoming the barrier of having limited money for class activities (e.g., clickers) shows no direct relationship with overcoming the barrier of limited time. However, in the case of supports (Fig. 2a), faculty can connect with supportive initiatives, such as national networks of EBT faculty and access online teaching resources, becoming part of a virtual community of practice that can cross institutional boundaries. Tapping into one support provides access to many.

### Practical implications

With regards to faculty training efforts, we suggest that the identification of supports be included and amplified during the EBT training at programs like the Summer Institutes for Scientific Teaching. Incorporating the new ideas introduced during such trainings may be challenging for faculty (e.g., restructuring a course, designing group activities). Such changes require effort and this effort may seem greater if faculty do not believe they will be supported by their department or if they believe their students will react poorly (Brew & Mantai, 2017; Henderson & Dancy, 2007; Michael, 2007). The theory of planned behavior suggests that these social norms influence faculty’s intention to incorporate EBT in their classrooms and is vital to consider if faculty’s teaching practice is to maintain change. Successful training could increase their effectiveness by anticipating these barriers and reacting to faculty’s concern by actively helping them identify resources, build supportive social connections, and establish a plan to integrate small, incremental EBT into their practice. In this way, training programs could align themselves with the finding that supports are important by increasing the attention they pay to them.

### Limitations

Our data is limited in the following ways, each of which is discussed in detail along with future directions from this work: (1) Our data is correlational in nature and causality cannot be assumed; (2) This sample is highly self-selected. Faculty here selected to attend a week-long training on EBT. We cannot assume that these patterns necessarily reflect the general faculty population; (3) Our study did not encompass all the variables that could explain variance in faculty’s decisions to implement EBT; (4) We relied fully on self-report variables; (5) This data examines the reported presence or absence of each EBT practice but does not address the frequency or quality of implementation, which presumably varies in the sample; and (6) We examined the relationship among supports, barriers, and EBT based on sum scores, which did not weight particular supports or barriers as more or less influential to reported implementation. It is likely that there are elements, such as a particularly strong barrier, that are more influential to teaching decisions than others.

### Future directions

Our data show a strong connection between perceiving support and reported implementation of EBT practices, but the direction is not causal. It could be that perceiving more resources *causes* faculty to more readily adopt EBT or it could be that faculty begin to perceive more supports *once they begin implementing* EBT (e.g., enjoyment, student responding well). While either direction is plausible, we posit that there is a *reciprocal* relationship between supports and implementation: The more supports that are perceived, the more EBT is implemented, which in turn, reinforces and increases the number of perceived supports. As such, training in EBT should have a dual focus. First, it should draw faculty’s attention to resources that encourage their implementation by helping them identify resources from within each of the support categories. Second, based on the idea that supports and implementation have a reciprocally reinforcing relationship, training should encourage faculty to begin implementing (if even in small ways) to help generate supports.

We show that perceptions of supports are strongly related to greater reported implementation and that barriers play a subdued role in comparison by using a large sample of college STEM faculty from across the nation. The faculty attending the SI training are likely highly motivated to change their teaching, as they are self-selecting to attend such a training, and may not fully represent the full spectrum of science faculty. Future research can build on this work and explore these patterns across different populations (e.g., less self-selected faculty, faculty untrained in EBT) and contexts (e.g., community colleges, K-12).

Incorporating EBT practices into teaching is a process (Aragón et al., 2017; Couch et al., 2015), and our current work is a snapshot into a progression that likely varies across individuals. Our model explained a significant and considerable amount of variation in faculty’s reported implementation, but there are additional individual and contextual factors contributing to how much faculty implement EBT. For example, the level of engagement in the EBT training, familiarity and confidence in teaching particular course content, and prior experience with EBT all likely contribute to faculty’s implementation. Future longitudinal work examining faculty’s adoption of EBT over time would provide insight as to how implementation changes following training and, along with it, perceptions of related supports and barriers.

While our study focuses on *perceptions* of supports and barriers rather than an objective coding of these features at specific institutions, we argue that what matters most to faculty’s implementation are exactly these perceptions. For example, if faculty members’ colleagues are utilizing EBT, but there is no discussion of its use, faculty are unaware of it and cannot leverage colleagues’ experience or resources (i.e., there is no support derived from it). Similarly, barriers are subjective. If there is only one faculty member advocating for EBT on their campus, some faculty may perceive this as a barrier, where others see an opportunity for departmental growth.

There are also supports and barriers that are more subjective by nature, such as personal teaching preferences (e.g., a faculty member’s comfort with implementing EBT). Reported implementation could also be verified more objectively, as well as deeper consideration about the specific practices used and the quality and frequency of the practices. Quality and frequency are likely strong moderators of the effect EBT practices may have on learning and should be included in this line of research. For example, there may be faculty who implement a few practices very effectively in a way that changes the culture of a course without needing to implement all the practices. Additionally, there may be practices that are used infrequently but with strong effects, such as setting expectations at the start of the course that can help align faculty and student expectations for what the course requires and provides.

The purely self-report data used here does limit our knowledge of the objective number of practices teachers are implementing and the supports and barriers that exist on campuses of which faculty may be unaware. There may be some discrepancy between what faculty are reporting and implementing and how frequently they use EBT practices, as has been reported in previous work using a similar sample (Ebert-May et al., 2011). It is challenging to fully observe teaching practices, as some EBT practices occur infrequently but serve to characterize a course. Stains et al. (2018) found that four observations of teaching were necessary to effectively describe the instructors’ teaching, more observations than what has previously been used to define gaps between reported and observed implementation (e.g., Ebert-May et al., 2011). To capture observational coding of our sample would require a substantially different study design than the largely exploratory one pursued here. The benefit of the current self-report method affords us the opportunity to reach hundreds of college science instructors who are SI alumni within a short period of time and administer a range of assessments beyond observations of their teaching. Next steps could be informed by knowing how these perceptions line up with observational coding. Having a mixed-method approach to understand actual versus reported implementation and the objective supports and barriers available to faculty across different campuses could direct research and interventions to better equip faculty to implement EBT (e.g., making faculty aware of supports vs. creating supports on their campus).

Finally, our work examines the sum of resources and challenges faculty perceive. This approach provides an overview of the broader relationship of supports and barriers to implementation of EBT but does not examine the relationship between particular features. For example, there may be barriers that are especially detrimental to implementation. Some barriers may passively prevent the use of EBT where others actively suppress it. For example, not having resources for active learning may prevent faculty from being able to readily make changes, but having a department that discourages EBT would be actively detrimental. There may also be particular supports that are most beneficial to overcome particular types of challenges. Relationships among specific resources and challenges are likely highly varied and contextualized to each faculty members’ teaching and home campus. Additional work examining these interactions among particular supports and barriers could suggest the most powerful areas to leverage interventions. We are currently building towards answering this question, as well as examining how particular types of EBT, such as inclusive teaching practices, may be differentially related to specific supports or barriers. Nonetheless, the present findings show a general approach of amplifying supports that would likely provide a boost in incorporating more EBT into science education.

## Conclusion

Overall, this work points to the importance of empirically considering the personal and contextual factors that support or hinder evidence-based teaching (EBT) implementation in college STEM classrooms using the theory of planned behavior as a framework. These factors, such as social support networks, access to resources, and personal preferences, are associated with reported EBT in somewhat unexpected ways. Our major finding that faculty’s reported implementation of EBT relates more strongly to the supports they perceive than the barriers they perceive warrants deeper study. This finding encourages a direction of research towards better understanding the relationship and function of supports and EBT implementation. In this way, we can learn to best leverage supportive features to promote greater adoption of EBT across the college STEM landscape.

## Additional file

Additional file 1: **Figures S1.** a and b show the correlation matrices for the support and barrier items. Darker colors indicate greater correlation. Bolded items are significantly correlated with each other at (minimally) a significance value of less than 0.05, with most being lower than .01. **Table S1.** Mapping of implementation items to the taxonomy in Couch et al. (2015). (DOCX 408 kb)

## Abbreviations

EBT: Evidence-based teaching
SI: Summer Institutes on Scientific Teaching
STEM: Science, technology, engineering, and math

## Footnotes

1 These evaluation surveys were collected via participant email directly following the SI training and the reviewed questions asked participants (a) “With regards to your home campus, please identify any factors that will support the implementation and dissemination of what you learned and developed at the Summer Institute” and (b) “Please identify any factors that will pose challenges to implementation and dissemination on your home campus.”

2 While our data does not distinguish whether instructors initially learned about these practices from other training programs, colleagues, or readings, each of these practices is clearly addressed in the SI curricula so that each instructor will be familiar with the content of our items.

## References

[CR1] Ajzen I (1985). From intentions to actions: A theory of planned behavior. Action control.

[CR2] Ajzen I (2011). The theory of planned behaviour: Reactions and reflections. Psychology & Health.

[CR3] Aragón OR, Dovidio JF, Graham MJ (2017). Colorblind and multicultural ideologies are associated with faculty adoption of inclusive teaching practices. Journal of Diversity in Higher Education.

[CR4] Association of American Universities Undergraduate STEM Initiative. (2013). *Framework for systemic change in undergraduate STEM teaching and learning*. Washington, D.C.: Association of American Universities.

[CR5] Basile V, Lopez E (2015). And still I see no changes: Enduring views of students of color in science and mathematics education policy reports. Science Education.

[CR6] Bradforth SE, Miller ER, Dichtel WR, Leibovich AK, Feig AL, Martin JD, Bjorkman KS, Schultz ZD, Smith TL (2015). University learning: Improve undergraduate science education. Nature.

[CR7] Brew A, Mantai L (2017). Academics’ perceptions of the challenges and barriers to implementing research-based experiences for undergraduates. Teaching in Higher Education.

[CR8] Brownell SE, Tanner KD (2012). Barriers to faculty pedagogical change: Lack of training, time, incentives, and … tensions with professional identity?. CBE—Life Sciences Education.

[CR9] Cavanagh Andrew J., Aragón Oriana R., Chen Xinnian, Couch Brian A., Durham Mary F., Bobrownicki Aiyana, Hanauer David I., Graham Mark J. (2016). Student Buy-In to Active Learning in a College Science Course. CBE—Life Sciences Education.

[CR10] Corbo, J. C., Reinholz, D. L., Dancy, M. H., Deetz, S., & Finkelstein, N. (2016). Framework for transforming departmental culture to support educational innovation. *Physical Review Physics Education Research, 12*(1), 010113.

[CR11] Couch BA, Brown TL, Schelpat TJ, Graham MJ, Knight JK (2015). Scientific teaching: Defining a taxonomy of observable practices. CBE—Life Sciences Education.

[CR12] Ebert-May D, Derting TL, Hodder J, Momsen JL, Long TM, Jardeleza SE (2011). What we say is not what we do: Effective evaluation of faculty professional development programs. BioScience.

[CR13] Fairweather, J. S., & Paulson, K. (2008). The evolution of scientific fields in American universities: Disciplinary differences, institutional isomorphism. In J. Valimaa & O. Yijoki (Eds.), *Cultural perspectives in higher education* (pp. 197–212). Dordrecht: Springer.

[CR14] Freeman S, Eddy SL, McDonough M, Smith MK, Okoroafor N, Jordt H, Wenderoth MP (2014). Active learning increases student performance in science, engineering, and mathematics. Proceedings of the National Academy of Sciences.

[CR15] George D, Mallery M (2010). SPSS for windows step by step: A simple guide and reference, 17.0 update.

[CR16] Greeno JG, Engeström Y, Sawyer RK (2014). Learning in activity. The Cambridge handbook of the learning sciences.

[CR17] Gross, D., Pietri, E.S., Anderson, G., Moyano-Camihort, K., & Graham, M.J. (2015). Increased preclass preparation underlies student outcome improvement in the flipped classroom. *CBE—Life Sciences Education, 14*, ar36. 10.1187/cbe.15-02-0040 PMC4710397 26396151

[CR18] Handelsman J, Ebert-May D, Beichner R, Bruns P, Chang A, DeHaan R, Gentile J, Lauffer S, Stewart J, Tilghman SM, Wood WB (2004). Scientific teaching. Science.

[CR19] Handelsman J, Miller S, Pfund C (2007). Scientific teaching.

[CR20] Henderson C, Dancy MH (2007). Barriers to the use of research-based instructional strategies: The influence of both individual and situational characteristics. Physical Review Special Topics—Physical Education Research.

[CR21] Herreid CF, Schiller NA (2013). Case studies and the flipped classroom. Journal of College Science Teaching.

[CR22] Hora MT (2012). Organizational factors and instructional decision-making: A cognitive perspective. Review of Higher Education.

[CR23] Lattuca LR, Bergom I, Knight DB (2014). Professional development, departmental contexts, and use of instructional strategies. Journal of Engineering Education.

[CR24] Lazerson M, Wagner U, Shumanis N (2000). What makes a revolution? Teaching and learning in higher education, 1980-2000. Change.

[CR25] Lee JJ (2007). The shaping of the departmental culture. Journal of Higher Education Policy and Management.

[CR26] Lund TJ, Stains M (2015). The importance of context: An exploration of factors influencing the adoption of student-centered teaching among chemistry, biology, and physics faculty. International Journal of STEM Education.

[CR27] Michael J (2007). Faculty perceptions about barriers to active learning. College Teaching.

[CR28] Moss-Racusin CA, Dovidio JF, Brescoll VL, Graham MJ, Handelsman J (2012). Science faculty’s subtle gender biases favor male students. Proceedings of the National Academy of Sciences.

[CR29] National Research Council. (2003). *Bio2010: Transforming undergraduate education for future research biologists*. Washington (DC): National Academies Press. 20669482

[CR30] Pfund C, Miller S, Brenner K, Bruns P, Chang A, Ebert-May D, Fagen AP, Gentile J, Gossens S, Khan IM (2009). Summer institute to improve university science teaching. Science.

[CR31] President’s Council of Advisors on Science and Technology (2012). Engage and excel: Producing one million additional college graduates with degrees in science, technology, engineering, and mathematics. Report to the President.

[CR33] Shadle SE, Marker A, Earl B (2017). Faculty drivers and barriers: Laying the groundwork for undergraduate STEM education reform in academic departments. International Journal of STEM Education.

[CR34] Stains M, Harshman J, Barker MK, Chasteen SV, Cole R, DeChenne-Peters SE (2018). Anatomy of STEM teaching in North American universities. Science.

[CR35] Vygotsky LS, Cole M, John-Steiner V, Scribner S, Souberman E (1978). Mind in society: The development of higher psychological processes.

[CR36] Walczyk JJ, Ramsey LL, Zha P (2007). Obstacles to instructional innovation according to college science and mathematics faculty. Journal of Research in Science Teaching.

[CR37] Wieman CE (2014). Large-scale comparison of science teaching methods sends clear message. Proceedings of the National Academy of Sciences.

[CR38] Wieman CE (2017). Improving how universities teach science: Lessons from the science education initiative.

