# Supplementary material for: Perceived supports and evidence-based teaching in college STEM
Source: Int J STEM Educ. 2019 Apr 9;6(1):11. doi: 10.1186/s40594-019-0166-3 (PMC6456468; doi:10.1186/s40594-019-0166-3)
Supplement: Supplementary file 1 — Figures S1. a and b show the correlation matrices for the support and barrier items. Darker colors indicate greater correlation. Bolded items are significantly correlated with each other at (minimally) a significance value of less than 0.05, with most being lower than .01. Table S1. Mapping of implementation items to the taxonomy in Couch et al. (2015). (DOCX 408 kb) [file 40594_2019_166_MOESM1_ESM.docx]

Additional file 1

Figures S1a and S1b show the correlation matrices for the support and barrier items. Darker colors indicate greater correlation. Bolded items are significantly correlated with each other at (minimally) a significance value of less than 0.05, with most being lower than .01.


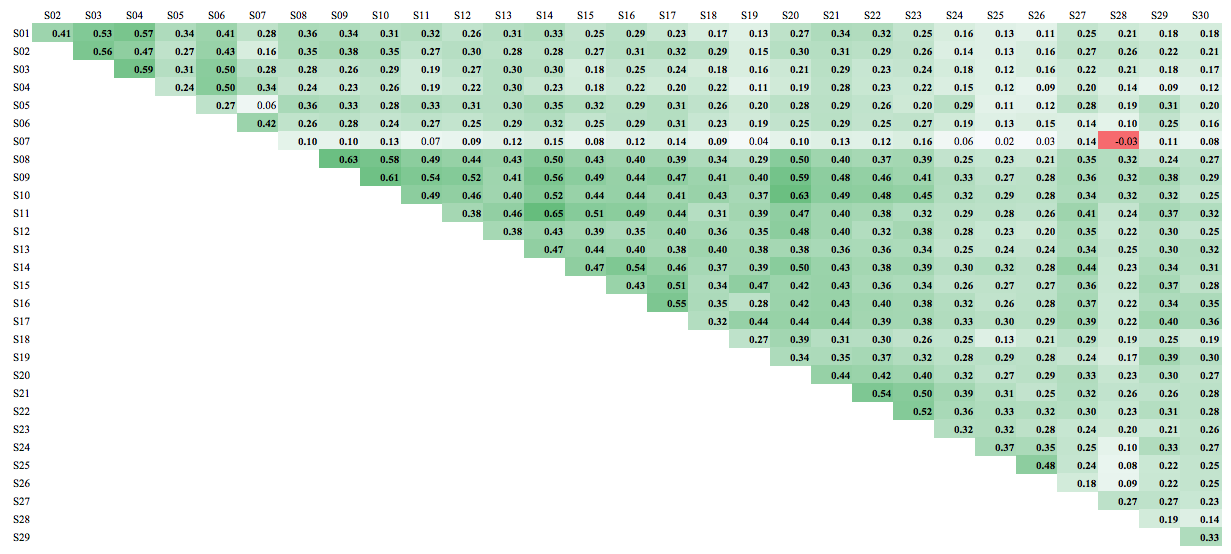


*Figure S1a*. Correlations among support items


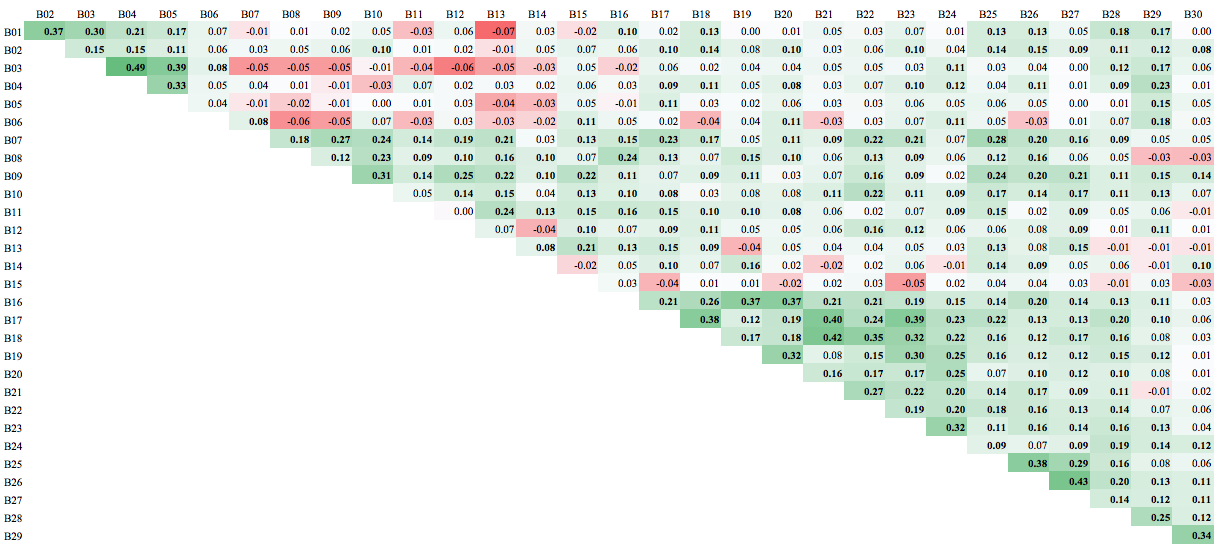


*Figure S1b*. Correlations among barrier items.

| **Implementation items** | **Relationship to Taxonomy** |
| --- | --- |
| Structuring class time to include activities that engage students in their own learning | 20, 36-37 |
| Providing feedback to students throughout the semester | 21, 23, 25 |
| Using exercises that generate group discussion | 22, 24-27 |
| At the onset of a course telling students what they should know and be able to do upon course completion | 1-2 |
| Considering learning goals in the design of activities for the class (backward design) | 1-3 |
| Using summative assessments of learning outcomes (i.e., to measure the students’ achievement of learning goals) | 3 |
| Implementing formative assessments while learning is occurring that inform students’ progress toward desired outcomes | 2, 21, 23, 25 |
| Representing science as a process of the scientific method | 10-19 |
| Setting and communicating learning goals for students for each class | 1-3 |
| Identifying students’ misconceptions so that they may be corrected | 7, 21, 25 |
| Using Blooms taxonomy which defines depths of understanding when preparing exams | 4, 32-34 |
| Using exercises that lead students to draw their own conclusions | 11, 15-17, 20, 34 |
| Encouraging students to think of science within the context of society | 8-10, 28 |
| Choosing diverse teaching methods to optimize learning for diverse students | 4, 6, 30 |
| Encouraging students to generate class wide discussions | 26 |
| Implementing inclusive teaching in the classroom | 28-31 |
| Taking precautions to reduce the influence of any implicit bias that I may hold for example grading papers without knowing the identity of the student | 31 |
| Encouraging students to think about their own learning processes aka metacognition | 36-37 |
| Designing class content that represents the perspectives and contributions of people with different origins genders and affiliations | 28-29 |

Table S1. Mapping of Implementation Items to the Taxonomy in Couch et al (2015).

*Note.* Relationship to taxonomy numbers reflect the supporting practice specified in Table 3 of Couch et al (2015). There are some practices in the taxonomy that indirectly relate these practices. Only those which are most clearly aligned are included in this Table. For example, many of these practices, such as providing opportunities for students to provide feedback on the course (Taxonomy item 5) could be considered inclusive teaching but only Taxonomy practices that explicitly encompass these topics are included in the table.
